# The Assessment of Risk and Predictors of Sleep Disorders in Patients with Psoriasis—A Questionnaire-Based Cross-Sectional Analysis

**DOI:** 10.3390/jcm10040664

**Published:** 2021-02-09

**Authors:** Julia Nowowiejska, Anna Baran, Marta Lewoc, Paulina Grabowska, Tomasz W. Kaminski, Iwona Flisiak

**Affiliations:** 1 Department of Dermatology and Venereology, Medical University of Bialystok, Zurawia 14 St, 15-540 Bialystok, Poland; anna.baran@umb.edu.pl (A.B.); lewocmarta@gmail.com (M.L.); paulina.dluzniewska29@gmail.com (P.G.); iflisiak@umb.edu.pl (I.F.); 2 Pittsburgh Heart, Lung and Blood Vascular Medicine Institute, University of Pittsburgh, Pittsburgh, PA 15260, USA; kamins1@pitt.edu

**Keywords:** sleep disorders, psoriasis, obstructive sleep apnea syndrome, restless legs syndrome, Pittsburgh sleep quality index, PSQI, STOP BANG, RLS

## Abstract

Psoriasis is a chronic, inflammatory skin disease affecting 2–4% of the general population. Accompanying subjective symptoms (pruritus or pain) may cause decreased life quality including sleep disorders (SD). Sixty psoriatic patients fulfilled the following questionnaires: Pittsburgh Sleep Quality Index (PSQI), STOP BANG for the obstructive sleep apnea syndrome (OSAS) assessment, and Restless Legs Syndrome (RLS) Severity Scale. Patients’ laboratory and clinical data were also investigated. All data obtained were compared to 40 participants without dermatoses. Mean PSQI, risk of OSAS, and RLS severity of psoriatics were significantly higher than in controls (*p* < 0.0001, *p* < 0.05, *p* < 0.05 respectively). There was a positive correlation between the time of suffering from psoriasis and the risk of OSAS (R = 0.286, *p* < 0.05). We did not observe any significant relationship between PSQI, risk of OSAS, or RLS and psoriasis severity assessed with PASI (Psoriasis Area and Severity Index). We identified four possible predictors of RLS: glucose, CRP and total cholesterol concentrations, and PSQI. SD are significantly more frequent in psoriatics than in people without chronic dermatological diseases but surprisingly they are not correlated with psoriasis severity. SD decrease patients’ life quality and may result in serious consequences. Therefore, establishing recommendations concerning screening for SD and their predictors should be considered.

## 1. Introduction

Sleep is an essential physiological activity that is dependent on homeostatic sleep drive and circadian rhythm [1]. Sleep and skin are bi-directionally related to each other because skin functions, such as thermoregulation and control of core body temperature, affect sleep course and the other way around—sleep disorders (SD) in patients with skin diseases may influence patients’ life quality and mental health or even exacerbate dermatological symptoms [2].

Psoriasis is a chronic, autoimmune, inflammatory skin disease affecting 2–4% of people worldwide [3,4]. It is a significant health and social issue because of decreased life quality and life expectancy five years shorter when compared to healthy persons due to comorbidities [3,5,6]. In our previously published papers, we have supported the current perception of psoriasis as a systemic disease of great impact on medical and psychological morbidity [7,8]. Psoriasis has been linked to coronary artery disease (CAD), arterial hypertension, diabetes mellitus (DM), obesity, and metabolic syndrome (MS) [3,9]. Numerous studies also have shown that psoriatics abuse alcohol and suffer from depression more often [3]. Furthermore, their life quality is worsened and comparable with persons suffering from chronic internal diseases, e.g., arterial hypertension, DM, inflammatory joint diseases, or malignant neoplasms [5,10].

There are multiple possibilities for which psoriasis could be associated with SD. Considering psoriasis is characterized by the presence of erythematous-papular lesions and plaques, which sometimes result in subjective symptoms such as pruritus or even pain, which can cause trouble with falling asleep or awakenings during sleep, it seems probable that psoriatics would also experience SD and have decreased sleep quality [2]. Additionally, depression itself, common in psoriatics, is frequently associated with impaired sleep, usually insomnia [10]. Moreover, it was proved that SD are more frequently observed in patients with heart failure, chronic obstructive pulmonary disease, or psoriatic arthritis (PsA), which are conditions also related to psoriasis [1,4,11,12]. Worse sleep quality was revealed in systemic lupus erythematosus which has autoimmune pathogenesis and skin involvement, similarly to psoriasis [13].

Obstructive sleep apnea syndrome (OSAS) is a sleep disorder that occurs with an uncertain frequency which varies depending on the source of information: from about 2–4% of the population [14] to even 20% of at least mild OSAS [15], with sleep breathing-related disorders, in general, reported affecting approximately 50% of the population which depends on sex and age of analyzed individuals [16]. It is characterized by episodes of recurrent upper airway collapse which lead to hypoxia, hypercapnia, and changes in the intrathoracic pressure [17]. OSAS is associated with the increased nocturnal activity of the sympathetic nervous system which results in elevated blood pressure and inflammatory and oxidative stress markers. All of these factors affect the cardiovascular system and can lead to serious complications [18]. In the clinical matter, OSAS manifests as apneas, hypopneas, loud snoring, awakenings, and daytime sleepiness [14,17]. Conditions that are the main risk factors of OSAS are, among others, obesity and type 2 DM, which are also closely related to psoriasis [3,17]. Moreover, oxidative stress and inflammatory processes are involved in the pathogenesis of OSAS, but also psoriasis [6]. OSAS was found to be linked to other autoimmune diseases such as lupus erythematosus and rheumatoid arthritis [19]. These findings, common risk factors, and pathomechanism suggest psoriatic patients might be at greater risk of OSAS. The estimated frequency of OSAS in psoriatics ranges from 13.7% to even 61.4%, which underlines the significant relationship between both diseases and the need for further research [14].

Restless legs syndrome (RLS) is another SD that nowadays gains more attention. Some studies indicate that it occurs more frequently in patients with autoimmune diseases, such as rheumatoid arthritis or lupus erythematosus [20]. There is also a strong relationship between RLS and systemic inflammation. The literature data show that nearly 90% of the medical conditions proved to be associated with RLS are inflammatory or infectious [21]. Considering similar, inflammatory, and autoimmune pathogenesis of psoriasis, there is also a possibility it favors RLS incidence. Furthermore, there is evidence that RLS is associated with cardiovascular diseases (CVD) and DM, which are more often observed in psoriatics [9,22]. Another possible link between RLS and psoriasis might be iron deficiency noted sometimes in both diseases [20,23]. According to the paper by Allen, the pathogenesis of RLS is not linked directly to the peripheral iron status but is more associated with iron deficiency in the central nervous system [24]. Moreover, most patients with RLS do not present abnormal ferritin concentration in serum and therefore it does not seem probable that the peripheral iron stores are insufficient [24]. The incidence of RLS in psoriatic patients has been previously investigated, although it requires further in-depth research due to discrepancies in outcomes. Some of these studies claimed RLS occurs more frequently [20,25,26] in patients with this dermatosis and others claim it does not occur significantly more often [27].

All of SD are proved to decrease patients’ quality of life, which in psoriatics is already decreased by the dermatosis itself [28]. SD have been reported to be associated also with increased risk of depression and anxiety, which in psoriatics may be additionally intensified [29]. Moreover, SD, OSAS in particular, are conditions leading to increased risk of CAD and car accidents [17,30]. SD have been quite unappreciated and insufficiently considered in daily practical psoriasis management so far. Considering their significant impact on psychophysical conditions they should be investigated more widely to provide their assessment in psoriasis management guidelines. Our aim was to assess sleep quality and the incidence and risk of SD, particularly OSAS and RLS in patients with psoriasis in association with disease severity, clinical and laboratory data, including inflammatory or metabolic disorders indices, and administered treatment, along with identification of possible SD predictors.

## 2. Materials and Methods

Sixty adult Caucasian patients with plaque psoriasis, hospitalized at the Department of Dermatology, fulfilled at admission a set of multiple validated surveys regarding their sleep patterns. Psoriasis severity was assessed by the same dermatologists using PASI (Psoriasis Area and Severity Index). Following exclusion criteria were set: pregnancy, malignant neoplasms, kidney diseases, thyroid diseases, infectious diseases, Parkinson’s disease, anemia due to iron deficiency, depression, anxiety disorders, and usage of particular medications (e.g., tricyclic antidepressants, pseudoephedrine, psychostimulants, opioids, lithium, theophylline, antiepileptics, and antidopaminergic agents, chronically used antihistamines). Every volunteer signed informed written consent before the enrollment and the study was approved by the local bioethical committee (No R-I-002/315/2018). The study was conducted according to the principles of the Declaration of Helsinki.

Questionnaires used to assess patients’ sleep involved: Pittsburgh Sleep Quality Index (PSQI), STOP BANG, and Restless Legs Syndrome Severity Scale. Pittsburgh Sleep Quality questionnaire consisted of 10 questions, which allowed for counting global PSQI. Global PSQI score > 5 meant poor sleep quality. The higher index, the poorer the sleep quality. Moreover, we adapted 4 questions from this questionnaire for a separate assessment, assigning from 1 to 4 points for each answer: the higher score, the more severe symptoms (these were sleep medicines usage, daytime dysfunction, and daily activities energy impairment) and for subjective sleep assessment—the higher score, the better sleep quality. STOP BANG questionnaire (Snoring, Tired, Observed, Pressure, BMI, Age > 50 years, Neck size large, Gender—male) consisted of 8 questions with answers ‘yes’ or ‘no’. Obtaining a particular number of ‘yes’ answers or presenting factors increasing independently risk of OSAS put every person into the group of low, intermediate, or high risk of OSAS. Patients who answered positively at least 2 of the 4 questions and who presented at least one of the following factors: male sex, BMI > 35 kg/m^2^, or neck circumference ≥43 cm (≥41 cm in females) were also classified as high-risk individuals. Neck circumference was measured using a tape measure.

RLS was diagnosed based on International RLS Study Group (IRLSSG) diagnostic criteria. Then, its severity was assessed with an RLS severity scale with a maximum score of 40 points. The higher score, the more severe RLS symptoms. Furthermore, patients were divided into two subgroups considering psoriasis severity evaluated with PASI: PASI I—mild psoriasis (PASI < 10), which consisted of 23 patients, and PASI II—moderate to severe psoriasis (≥10) of 37 patients. Another subdivision included two subgroups: one treated only with topical agents (25 individuals), and the second was also treated with systemic drugs (35 individuals: 15 with methotrexate, 16—acitretin, 4—cyclosporine A). Additionally, we divided the study group regarding age: under 45 years old (20 individuals) and over 45 years old (40 individuals). Another division was into two subgroups of patients taking (17 individuals) and not taking sleeping medicines (43 individuals). Moreover, the medical history and laboratory parameters of patients were investigated. All data obtained were compared to the sex- and age-matched control group of 40 volunteers (21 males, 19 females) without dermatological disorders.

All the data were subjected to cross-sectional statistical analysis. The normality of distribution was tested using the Shapiro–Wilk test and normally distributed data were expressed as mean ± SD. The not normal distributed data were presented as median (minimum-maximum). The Student’s t-test or nonparametric Mann–Whitney test were used to compare differences between two groups, whereas, for gender diversity and RLS occurrence, Chi-square test was used. The correlations between studied variables were determined by Spearman’s rank correlation analysis. A multivariate multiple regression model has been used to detect the predictors of RLS. Variables considered in this study were selected based on the literature and our clinical experience. Potential determinant factors expected to be correlated with SD among patients are included as variables of the study. A two-tailed *p*-value < 0.05 was statistically significant. The power of statistical analysis was estimated using StatMate2.0 software (GraphPad Software; La Jolla, CA, USA). The number of enrolled patients was estimated based on literature and preliminary data, and assuming a power of 0.8 to detect a difference of at least 20% with a significance of 0.05. Computations were performed using GraphPad 7 Prism Software (GraphPad Software; La Jolla, CA, USA).

## 3. Results

Baseline characteristic of patients and controls is present in Table 1.

The patients’ group consisted of 31 men and 29 women, mean age was 49.75 ± 17.03 years old. Median BMI was 25.45 (17.01–42.10) and compared to controls, there were no significant differences. Median PASI before treatment was 14.23 (2–44.4), and after the treatment, it decreased significantly (*p* < 0.0001) to 8.6 (0–25). After division into two age subgroups, we noticed higher levels of glucose (*p* < 0.05) and uric acid (*p* < 0.01) concentrations in the mature group (Table 2). In the PASI II subgroup, significantly more patients suffered from PsA (*p* < 0.05) (Table 3).

### 3.1. Sleep Quality Assessment

**Figure 1:**
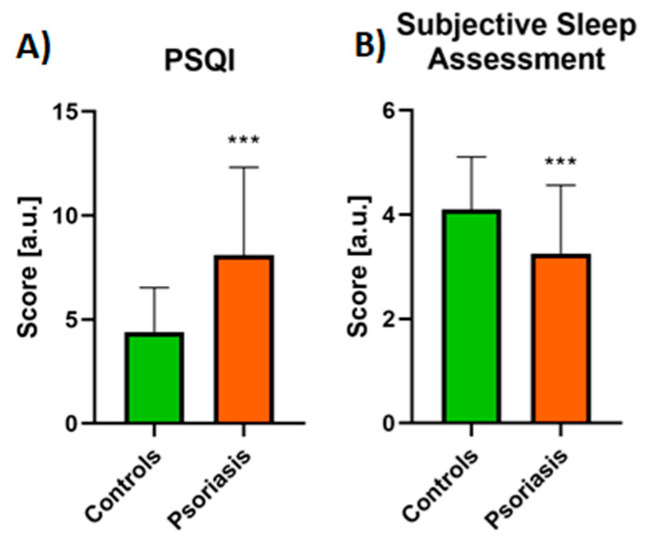
Assessment of PSQI (**A**) and subjective sleep assessment (**B**) in psoriatics and control groups. Graphs A-B present data shown as Mean ± SD and values are normally distributed. ***—means the existence of a statistically significant difference between values treatment with *p* < 0.0001. PSQI, Pittsburgh Sleep Quality Index. Data are shown as Mean ± SD or Contingency Graph.

Mean PSQI was 8.1 ± 3.22 and was significantly higher (*p* < 0.0001) than in controls: 4.4 ± 2.13 (Figure 1A).

PSQI above 5, which meant poor sleep quality, was noticed in 47 patients (78.3%), 24 patients (40%) subjectively assessed their sleep quality as fairly bad or bad. For comparison, only 6 individuals from the control group (15%) reported bad sleep quality, and none of them bad sleep quality. The difference in the subjective assessment of sleep quality between the studied groups was significant (*p* < 0.01, Figure 1B). We did not find any associations between PSQI and sex, PASI nor BMI scores, or with the duration of the disease. After division into two subgroups considering PASI, PSQI was higher in the PASI II subgroup although the difference was not significant (Table 3), same for the division into two age groups (Table 2).

**Figure 2:**
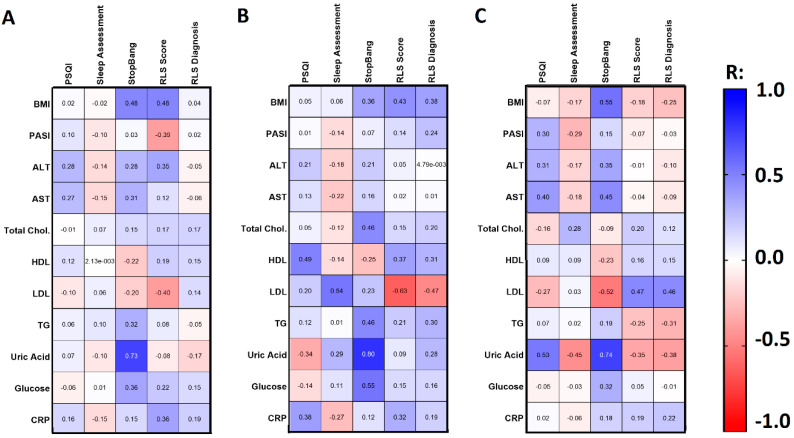
Spearman’s rank correlations between chosen basic clinical data and sleep patterns scores in psoriatic patients’ group (**A**), PASI I subgroup (**B**), and PASI II subgroup (**C**). Numbers on the graphs present R rank values. The blue color indicates the existence of a positive correlation between analyzed parameters, whereas the red color stands for the negative dependencies. BMI, body mass index; PASI, psoriasis area and severity index; TGs, triglycerides; HDL, high-density lipoproteins; LDL, low-density lipoproteins; CRP, C-reactive protein; ALT, alanine transaminase; AST, asparagine transaminase; PSQI, Pittsburgh Sleep Quality Index; RLS, restless legs syndrome.

In basic laboratory parameters, we observed a significant positive correlation between aminotransferases levels and PSQI (R = 0.28 for ALT, R = 0.27 for AST, Figure 2A).

These dependencies were also found in the PASI II subgroup (R = 0.312 and R = 0.397 respectively, Figure 2C). Patients on systemic treatment had significantly higher PSQI than those on topical treatment (*p* < 0.05, Table 4).

**Figure 3:**
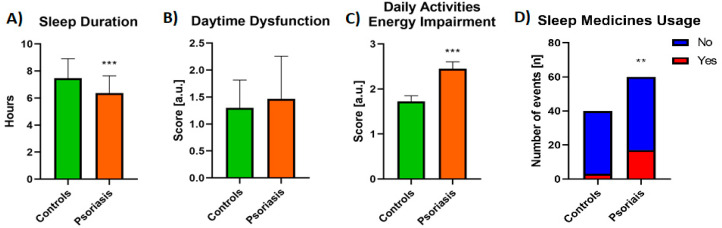
Differences in sleep duration (**A**), daytime dysfunction self-assessment (**B**), overall impairment of daily activities (**C**), and usage of sleep medicines (**D**) between controls and psoriatic patients. **/***—means the existence of statistically significant difference between values treatment with *p* < 0.01; <0.0001 respectively. Data are shown as Mean ± SD or Contingency Graph.

Patients reported a shorter sleep time than controls: the mean sleep duration was 6.37 ± 1.28 hours a day for patients and 7.46 ± 1.45 for controls, which was statistically different (*p* < 0.0001, Figure 3A). Over one-fourth of patients reported using sleep medicines (28.33%), among which the majority (18.3% of all patients) three or more times a week; comparing to controls of whom only three persons (7.5%) admitted taking sleeping pills, so the difference was significant (*p* < 0.01, Figure 3D). There was no statistically significant difference in comparison between the subgroups taking and not taking sleeping medicines, except for daytime dysfunction assessment which was even lower for individuals not using them (*p* = 0.267). Thirty percent of patients and 27.5% of controls reported daytime dysfunction, which means they had trouble staying awake while driving car/eating meals/social activities, and the difference between the groups was not significant (NS) (Figure 3B). 71.66% of patients and 55% of controls reported having less energy to perform daily activities (to a different extent) and the difference was significant (*p* < 0.01, Figure 3C.).

### 3.2. OSAS Assessment

**Figure 4:**
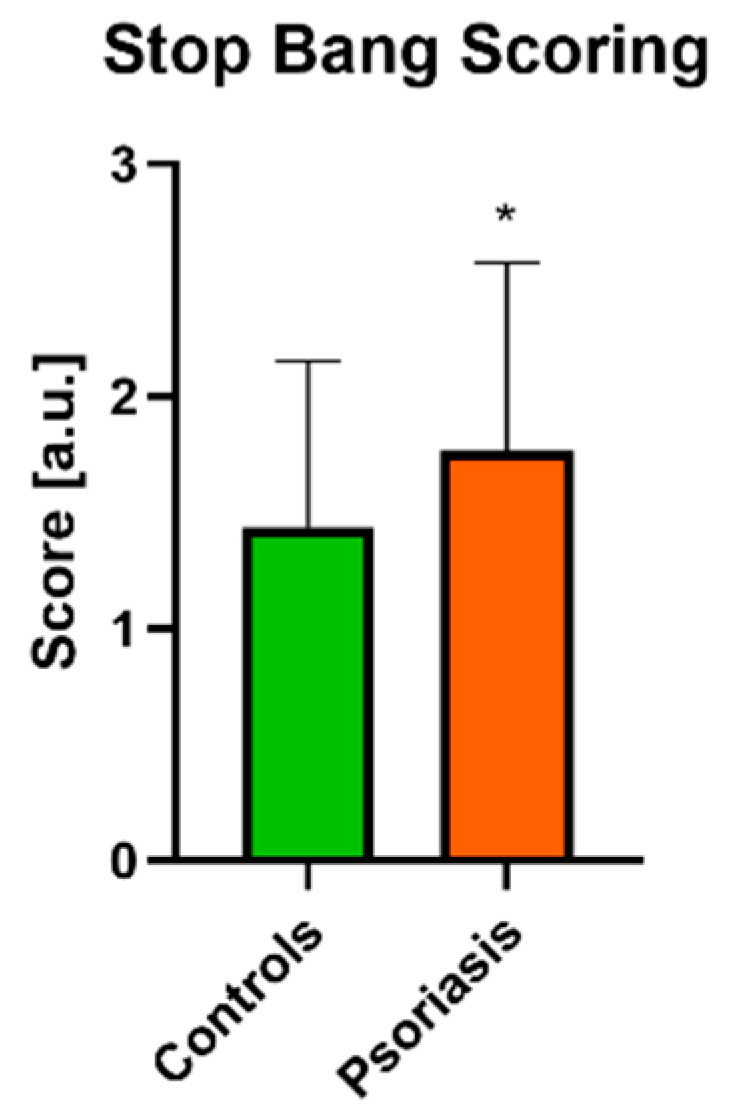
Assessment of risk of OSAS in psoriatics and control groups. The graph presents data shown as Mean ± SD and values are normally distributed. *—means the existence of a statistically significant difference between values treatment with *p* < 0.05. OSAS, obstructive sleep apnea syndrome. Data are shown as Mean ± SD or Contingency Graph.

High risk of OSAS according to STOP BANG was noted in 14 of 60 patients (23.3%)—12 males and 2 females, intermediate risk in 18 persons (30%)—7 males and 11 females, and low in 28 (46.6%)—12 males and 16 females. Increased risk of OSAS (high and intermediate) was significantly higher in patients than in controls (*p* < 0.05) (Figure 4).

We found that the older group of patients (over 45 years old) were at significantly higher risk of OSAS than the younger group (*p* < 0.01, Table 2). A positive correlation between the risk of OSAS and BMI was observed (R = 0.554) in PASI II, and a similar association has been found in PASI I (R = 0.359, Table 3). There was a significant correlation between the time of suffering from psoriasis and the risk of OSAS (R = 0.286). We observed no relationship between PASI and the risk of OSAS nor differences in OSAS scoring, even after division into two subgroups considering PASI. Of the laboratory investigations, we noticed a positive correlation between aminotransferases levels and the risk of OSAS, particularly in the PASI II subgroup (R = 0.354 for ALT and R = 0.451 for AST respectively, Figure 2C). We observed no correlations between the risk of OSAS and cholesterol levels in the patients in total (Figure 2A). Significant associations between OSAS and glucose (R = 0.36), triglycerides (R = 0.32) and uric acid (R = 0.73) levels have been noted though (Figure 2A), especially in the PASI I subgroup (Figure 2B), for which correlations with glucose (R = 0.55) and uric acid (R = 0.8) were noted, and moreover with total cholesterol and triglycerides levels (both R = 0.46, Figure 2B). We did not observe any influence of topical vs. systemic treatment on the risk of OSAS (Table 4). We did not find any correlation between the risk of OSAS and PSQI or RLS severity either.

### 3.3. RLS Assessment

**Figure 5:**
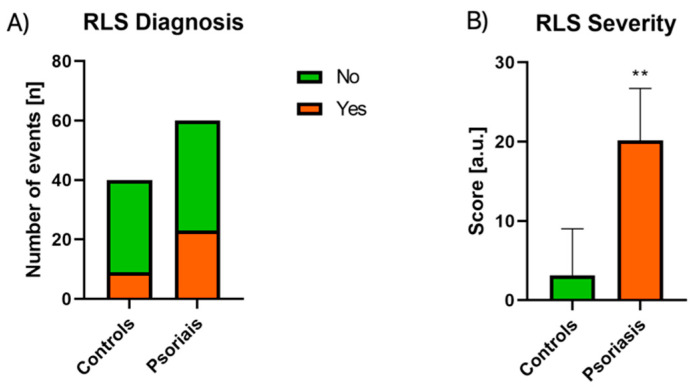
Assessment of frequency (**A**) and severity of RLS (**B**) in psoriatics and control groups. Graph B presents data shown as Mean ± SD and values are normally distributed. **—means the existence of a statistically significant difference between values treatment with *p* < 0.01. RLS, restless legs syndrome. Data are shown as Mean ± SD or Contingency Graph.

RLS was diagnosed in 23 of 60 patients (38.3%; 14 females and 9 males) comparing to controls: 9 of 40 individuals (22.5%), which was not significantly more frequent (Figure 5A).

On the other hand, the mean severity of RLS symptoms assessed on a 40-point scale was 20.13 ± 6.59, which was a significantly higher score (*p* < 0.01) compared to controls’ mean score of 13.78 ± 2.28 (Figure 5B). We found a correlation between sex and RLS severity (R = −0.283) and a trend for RLS diagnosis (R = −0.2). We did not observe any significant relationship between RLS severity and BMI, PASI, or laboratory parameters, except for a positive correlation with CRP levels (R = 0.363, Figure 2A). After division into two subgroups considering PASI, the RLS was more frequent in the PASI II subgroup although the difference was not significant (Table 3), same for the division regarding the age of patients (Table 2). We found no correlation between the duration of psoriasis and RLS diagnosis or severity. Regarding administered treatment, we found no significant differences in RLS diagnosis in terms of topical or systemic treatment or no correlation with any systemic agent in particular. We only observed increased RLS severity in patients on systemic treatment compared to patients undergoing topical pharmacotherapy (*p* < 0.05, Table 4). RLS severity was on the other hand positively correlated with PSQI (R = 0.342) but not with the risk of OSAS in STOP BANG. We found four parameters independently associated with RLS diagnosis, namely: total cholesterol, glucose, and CRP concentration as well as PSQI (Table 5).

## 4. Discussion

The importance of the sleep issue has been well visualized in Maslow’s hierarchy of needs presented as a pyramid consisting of five main categories of fundamental human needs. At the base of the pyramid, the most primitive, physiological needs are located, which are the most powerful among all at the same time. These are (besides food, water, and shelter) sleep and rest [31]. Therefore, the subject discussed in our study is extremely relevant since it concerns the most basic human needs which have to be satisfied, otherwise, people cannot function properly.

The SD issue in psoriatics has recently gained more and more attention [1,11,14,32,33] and the time we designed our study information regarding this topic was limited. Although it has been investigated and some publications provided data confirming worse sleep quality in psoriatic patients, there are still many unknowns and much to explain in this matter [3,32]. Indeed, also in our analysis, decreased sleep quality occurred significantly more frequently in psoriatics comparing to controls, with significantly higher PSQI. Psoriatic patients also reported to sleep significantly fewer hours than individuals in the control group and also subjectively assessed their sleep as worse. These results implicate that psoriatic patients are at high risk of SD and screening for poor sleep quality should be considered, especially it seems relatively easy to perform. We suggest every dermatologist treating a psoriatic patient should evaluate them regarding the sleep quality using one of the available validated screening questionnaires, as we did in this research. Currently, there are no guidelines regarding the sleep quality assessment among psoriatics in the American Academy of Dermatology—National Psoriasis Foundation (AAD-NPF) guidelines or other local ones [34,35]. There are different measures available to assess sleep quality. The most popular are Epworth Sleepiness Scale (ESS) and PSQI. ESS is a self-administered 8-item questionnaire used to assess daytime sleepiness by evaluating the likelihood of dozing during different activities [32]. In this study, we chose the latter scale as it has been proven to be of good reliability and validity for both healthy and clinical groups with mental and physical health disorders in different age groups, both young and elderly, and in different cultural backgrounds [36]. A global PSQI score of more than 5 has a diagnostic sensitivity of 89.6% and specificity of 86.5% in differentiating poor from good sleep quality [37]. PSQI regards various different sleeping habits of an individual assessed within the preceding month [32].

Importantly, there are inconsistent data available in the literature regarding whether deterioration of sleep quality correlates with the severity of psoriatic lesions in PASI. Stinco et al. claimed there was no correlation [38], on the other hand, Melikoglu et al. confirmed this relationship [33]. In our research sleep quality did not correlate with the severity of psoriasis evaluated with PASI. Therefore, we conclude PASI score cannot be used as a predictor of the decreased sleep quality and its severity in psoriatic patients. Moreover, every psoriatic, despite the disease severity, should be evaluated regarding SD, and patients with low PASI scores cannot be omitted assuming they are at less risk of bad sleep quality. We assume this issue is much more complex. Supposedly, there is a “vicious circle” between SD and skin lesions’ severity. Subjective symptoms released by psoriasis decrease everyday life quality and induce SD. Decreased sleep quality exacerbates stress and worsens even more life quality. Severe stress is well-known to worsen psoriatic skin conditions, which further decreases life and sleep quality [10]. It is worth considering that all of the mentioned relationships are not directly proportional. Moreover, PASI, despite being the most commonly used score in everyday practice, is a subjective scale and is burdened by the skills and experience of assessing physician [39]. Currently, there is no severity scale that could be used in psoriasis that would meet all the validation criteria required for an ideal score [40]. Although PASI cannot become a reliable marker of decreased sleep quality in psoriatics, there are data supporting worse sleep quality in patients with PsA comparing to individuals with only skin involvement [11].

We did not find any correlation between BMI and PSQI which implicates that decreased sleep quality affects all psoriatics, regardless of their body mass. BMI, similar to PASI, cannot serve as a predictor of decreased sleep quality in psoriatics. Literature data concerning these results are not consistent. In a study conducted by Tas et al. outcomes similar to ours were observed—PSQI is not correlated with BMI in psoriatics [41]. In other research, but not concerning psoriatics, some authors suggest that such a relationship exists [42], whereas some only noted such a relationship in women [43]. We also did not observe any significant relationship between PSQI and serum lipid levels or glucose as metabolic disorders indices, therefore these laboratory parameters do not seem to be useful in the prediction of worse sleep quality among psoriatics. On the other hand, in a large cohort study conducted by Geovanini et al., assessing sleep quality in PSQI and its associations with different cardiometabolic parameters, there was a significant correlation between higher PSQI (worse sleep quality) and abnormal lipid profile [44]. It was a diverse rural cohort though, and we did not observe such relationships particularly in psoriatics. Considering we found a positive correlation between PSQI and aminotransferases, particularly in the group of severe psoriasis, we suggest that these liver function markers may possibly serve as predictors of SD in psoriatic patients. Possibly, the higher the aminotransferase level, the worse sleep quality. Unfortunately, to the best of our knowledge, there are no studies regarding associations between PSQI and aminotransferases levels in psoriatics, so we have no data for comparison. Moreover, we should consider the possible influence of drugs on the results of aminotransferase activity. Thus, the outcomes obtained require further in-depth research. There was a significant difference in PSQI between patients treated with topical agents and systemic drugs. Patients who required systemic treatment had higher PSQI than those only on topical treatment, which seems reasonable and could be explained due to the fact of the necessity of administration of systemic treatment to patients with more severe psoriatic symptoms. Although we did not obtain a statistically significant correlation between psoriasis severity in PASI and PSQI, individuals with moderate to severe psoriasis had higher PSQI indeed. Similar data is lacking for a direct comparison, but one research assessing SD in patients with psoriasis and PsA revealed no improvement in sleep quality after methotrexate administration (similar to our outcomes), while it was observed after treatment with anti-TNF-alfa agents [11]. Similarly, in one research investigating sleep quality in patients with rheumatoid arthritis, parameters of sleep efficacy and awakenings improved after the introduction of TNF-alfa inhibitors [45]. We found no statistical differences between the patients’ sleep quality regarding the particular drug they were treated with. Continuing the drug issue, we observed significantly more frequent sleep medicine use in patients compared to controls. What we managed to confirm is eminently associated with significantly increased PSQI in this group. Furthermore, it indicates that psoriatics cannot easily cope with SD, which makes them take sleeping pills, mostly very often, which seems serious and highlights the importance of such disorders in psoriatics along with the need of prescribing such medicine to this group of patients. At the same time, there were no significant differences in the comparison between the subgroups taking and not taking sleeping medicines in our analysis.

Besides sleeplessness, some research shows that there is a greater risk of OSAS in psoriatic patients [14,46,47]. Publications vary in information whether it comes to the total risk of OSAS in psoriatics, stratification of this risk (exactly what we intended to investigate), or actual OSAS incidence. In our study we surprisingly observed that most of psoriatics have low risk of OSAS using the STOP BANG questionnaire, the same in the controls, although the proportions of each level of risk were in favor of a lower risk in the controls, which seems reasonable. Finally, we found a statistically significant difference in the overall increased (intermediate to high) risk of OSAS between the patients and controls. It clearly shows that OSAS screening is another activity that should be performed by physicians, particularly dermatologists, in their daily practice. Moreover, we noted that the longer patients suffer from psoriasis, the higher risk of OSAS they have. That implies that OSAS questionnaires should be introduced especially to the group of long-time psoriatic patients. Such screening is important since OSAS results in severe medical complications. It was proved that OSAS leads to an increased risk of cardiovascular diseases and may contribute to the same disorders as observed in MS [48]. Considering the already increased frequency of cardiometabolic diseases in psoriasis, it should be assumed that the coexistence of OSAS will additionally increase this risk. Similar to PSQI, we did not observe any correlation between the severity of skin lesions in PASI and the risk of OSAS, which means that all psoriatics are at greater risk of OSAS than controls, no matter how severe psoriasis is. Therefore, PASI cannot serve as a predictor of the risk of OSAS in psoriatics. Same as for PSQI, no patient, even despite a low PASI score, cannot be disregarded in relation to the risk of OSAS. We also did not find any significant superiority of systemic instead of topical treatment on OSAS risk, which possibly implicates that most commonly used antipsoriatic systemic drugs do not improve OSAS symptoms. On the other hand, in the end, proper, effective treatment improves the quality of life of psoriatics, reduces subjective symptoms (pruritus, pain), and thus should indirectly improve the quality of sleep. Therefore, further studies should be provided also involving psoriatics before and after the implementation of different therapeutic methods. To the best of our knowledge, this is the first observation regarding the influence of the therapy on SD in psoriatics. There are reports on a positive impact of TNF-alfa inhibitors on OSAS in patients with spondyloarthritis. Since the same agents are widely applied in psoriasis that may be a possible further research path [49]. An interesting finding was reported by Buslau et al. who noted an improvement of psoriatic skin condition after administration of nCPAP (nasal continuous positive airway pressure) to three patients with OSAS [50]. This highlights a close relationship between these two entities. Of the laboratory investigations, we observed a similar relationship as for PSQI: a positive correlation between the risk of OSAS and aminotransferase activity, particularly in the group of severe psoriasis. Since there is research investigating liver function enzyme activity in patients with OSAS [51], our outcome is consistent with other research and the observed higher aminotransferase activity in our psoriatic patients can be simply related to OSAS, with no impact on psoriasis. Individuals with OSAS had higher ALT and AST activity compared to controls and the possible explanation for this is hypoxia as a liver-damaging factor [51]. Moreover, the higher the activity, the more severe OSAS [51]. That is why these associations need to be further investigated, perhaps comparing the group of psoriatics with OSAS and individuals with OSAS alone. We also observed a positive correlation between OSAS risk and triglycerides, total cholesterol, glucose, and uric acid concentrations which parameters are known to be elevated in obese OSAS patients as well [52,53]. Obviously, we found a strong positive correlation between the risk of OSAS and BMI, which is consistent with the literature data considering obesity, especially visceral, and therefore high BMI, as one of the most important risk factors of OSAS [54]. It confirms that losing weight, which results in lowering BMI, decreases OSAS risk, which is provided by literature data. Among different weight-reduction interventions, not a single one of them has been established to be the most effective in all patients with OSAS and there is a need for further investigation [54]. Importantly, similar observation has been made in psoriatics. Losing weight helped in the reduction of skin lesions and decreasing of PASI [55]. Since we have matched the control group with the sex and age of the patients and the BMI was not statistically significantly different between both groups, we eliminated three main factors independently increasing the risk of OSAS in the STOP BANG questionnaire which makes our outcomes reliable.

Furthermore, there are some publications regarding the more frequent occurrence of RLS in patients with psoriasis, although there are some inconsistencies. In our study, symptoms of RLS did not occur significantly more frequently in psoriatics, but in our opinion, it requires further investigation because the assessment of RLS criteria and interviewing patients to detect RLS symptoms is difficult. RLS is often misunderstood by individuals which makes them assess their impressions often incorrectly. In our study, we encountered plenty of obstacles in this part of the research, especially due to misunderstanding by the patients that RLS is a particular disease, characterized by specified diagnostic criteria and they sometimes exaggerated their symptoms. Still, patients who actually met RLS criteria presented more severe symptoms of RLS than controls. Therefore, it suggests that if a psoriatic actually suffers from RLS, effective treatment of this dermatosis may probably ease RLS symptoms, and psoriasis itself may worsen the RLS course. RLS is reported to be more frequently observed and more severe in women, which we managed to note as well [56]. We did not observe a significant relationship between RLS severity and PASI, which suggests that all psoriatics are at risk of RLS no matter how severe the skin lesions they have are and PASI cannot serve as a marker of RLS risk. BMI was also not significantly associated with RLS severity which shows that the weight of patients does not influence RLS severity. We found four parameters independently correlated with RLS diagnosis which could be regarded as predictors of RLS: CRP, total cholesterol, glucose concentrations, and PSQI. Possibly CRP could serve as a marker of prediction of RLS occurrence and severity—the higher CRP, possibly the higher probability of RLS and its more severe symptoms. At the same time, there are studies assessing CRP concentration in RLS patients. One of them was performed to investigate CRP concentration in individuals with RLS in general and revealed its increased levels compared to controls [57]. On the other hand, we found another study that could be applicable to our findings, but it investigated possible correlation particularly between periodic leg movements (PLMs) of sleep in RLS and CRP concentration. The result was that patients with RLS, only those with high numbers of PLMs (i.e., at least 45/hour), were significantly more likely to have increased CRP levels [21]. Therefore, it requires further investigation whether RLS itself is related to higher CRP concentration or there are more variables affecting its serum levels. RLS severity was also found to be positively correlated with PSQI. It suggests that the more severe RLS symptoms, the worse sleep quality, which seems reasonable. At the same time, RLS severity was not correlated with the risk of OSAS in STOP BANG. It shows that these two disorders can appear independently. It seems explainable since we noticed a significant association only between psoriasis and the risk of OSAS, and not with the frequency of RLS. Both glucose metabolism disorders and elevated cholesterol levels have been reported to be associated with RLS, as we mentioned above, therefore our results seem reasonable and their concentrations in psoriatics could become predictors of RLS occurrence [56,58]. Moreover, considering both conditions (psoriasis and RLS) are related to carbohydrates disorders and hypercholesterolemia we advise blood glucose and cholesterol concentration monitoring in such patients, especially in case of their coexistence. The dependencies we found are a novel contribution to the current state of knowledge and suggest there might be a few parameters that should be further investigated on a larger scale as promising predictors of RLS.

It must also be taken into consideration that there are some other aspects influencing sleep and that it is affected by different psychological and environmental factors such as socioeconomic status [59,60]. There is also a reverse relationship, as sleep deprivation can lead to decreased quality of life, deterioration in energy to perform everyday activities and inability to work [61].

Considering the limitations of our study, it was a single-center research with a relatively small number of patients. Our group of patients was diverse in terms of PASI and results were obtained on the basis of the subjective assessment, therefore they may contain a flaw. In the future, we would like to extend our research in cooperation with other departments managing SD in order to perform polysomnography in psoriatic patients, as well as to correlate different parameters analyzed in this procedure with other variables presented in this paper.

## 5. Conclusions

The results revealed that SD are an essential problem in psoriatic patients. We have confirmed that psoriatics have decreased sleep quality, sleep fewer hours than individuals free from skin diseases, frequently take more sleep medicines, and have less energy in everyday activities. Psoriatics also present a higher risk of OSAS which increases along with the duration of the disease, and more severe symptoms of RLS, so we conclude that psoriasis may worsen the course of RLS. PASI is not correlated with sleep quality, OSAS risk, and RLS severity, therefore, it cannot serve as a predictor of SD in psoriatics. PSQI and RLS severity in psoriatics are also not dependent on BMI. Aminotransferase activity could serve as a predictor of decreased sleep quality in patients with moderate to severe psoriasis but the association with OSAS risk has to be further investigated. As for possible predictors of SD, we excluded different parameters as such markers, especially PASI which surprisingly cannot be used for the prognosis of SD. On the other hand, CRP, total cholesterol, and glucose concentrations may become predictors of RLS occurrence in psoriatics. Screening for described SD should be considered in all psoriatic patients using cheap and easy questionnaires, independently on PASI score and included in psoriasis management guidelines.

## Figures and Tables

**Table 1:** Baseline characteristics and comparison of patients and controls.

Parameter	Controls *n* = 40	Psoriatic Patients *n* = 60
Sex (M/F)	21/19	31/29 NS
Age (years)	49.78 ± 17.9	49.75 ± 17.03 NS
BMI	25.38 (20.01–35.5)	25.45 (17.01–42.10) NS
PASI before treatment	-	14.23 (2–44.4)
PASI after treatment	-	8.6 (0–25) ***

***—means the existence of statistically significant difference between values before and after treatment with <0.001, NS, non-significant. BMI, body mass index; PASI, psoriasis area, and severity index; Data is shown as Mean ± SD for normal distributed values or median (full range) for skewed distribution values.

**Table 2:** Division and comparison between two age groups of patients.

Parameters	Under 45 *n* = 21	Over 45 *n* = 39
Sex (M/F)	10/11	21/18 NS
BMI	24.03 (17.3–41.21)	26.75 (17.01–42.1) NS
PASI before	10.7 (4–27.3)	12.6 (2–44.4) NS
ALT	17 (6–77)	19 (6–98) NS
AST	24.38 ± 8.9	28.1 ± 13.01 NS
Total cholesterol	154.2 ± 36.76	159.2 ± 41.74 NS
HDL	39.71 ± 10.98	45.75 ± 15.28 NS
LDL	92 ± 31.96	90.55 ± 51.81 NS
TG	*119.6 ± 37.24*	*140 ± 70.99* NS
Uric acid	**4.41 ± 0.93**	6.46 ± 1.85 **
Glucose	**84.81 ± 13**	97.08 ± 32.89 *
CRP	*2.44 (0.66–46.2)*	*4.395 (1.03–91) NS*
PSQI	7.47 ± 4.4	8.43 ± 4.15 NS
Subjective sleep assessment	3.38 ± 1.28	3.18 ± 1.33 NS
STOP BANG	**1.28 ± 0.71**	2.04 ± 0.71 *
RLS diagnosis	6/14	17/22 NS
RLS severity	19.33 ± 8.52	20.41 ± 6.05 NS

*/**—means the existence of statistically significant difference between values treatment with *p* <0.05; < 0.01, respectively. *Curved font* means the existence of trend NS, non-significant. BMI, body mass index; PASI, psoriasis area and severity index; TGs, triglycerides; HDL, high-density lipoproteins; LDL, low-density lipoproteins; CRP, C-reactive protein; ALT, alanine transaminase; AST, asparagine transaminase; PSQI, Pittsburgh Sleep Quality Index; RLS, restless legs syndrome. Data is shown as Mean ± SD for normal distributed values or median (full range) for skewed distribution values.

**Table 3:** Comparison between two subgroups: PASI (Psoriasis Area and Severity Index) I and PASI II in terms of baseline data, standard laboratory parameters, and values of sleep patterns.

Parameter	PASI I *n* = 23	PASI II *n* = 37
Sex (M/F)	**8/15**	23/14 *
BMI	28.63 (18.11–41.21)	25.16 (17.01–42.1) NS
Psoriatic arthritis	**1/22**	9/28 *
ALT	17 (10–77)	20 (6–98) NS
AST	21 (10–100)	24 (12–81) NS
Total cholesterol	160.3 ± 38.41	155.7 ± 41.1 NS
HDL	43.11 ± 11.04	44.72 ± 16.01 NS
LDL	81 (41–197)	74 (24–154) NS
TGs	124.4 ± 53.12	138.1 ± 66.7 NS
Uric acid	4.6 (3.3–11.17)	6.1 (3.2–8.9) NS
Glucose	94.91 ± 28.81	91.46 ± 27.94 NS
CRP	4 (1.03–46.2)	3.66 (0.66–91) NS
PSQI	7.91 ± 4.06	8.22 ± 4.31 NS
Subjective sleep assessment	3.22 ± 1.13	3.27 ± 1.43 NS
STOP BANG	1.74 ± 0.81	1.78 ± 0.82 NS
RLS diagnosis	8/15	15/22 NS

*—means the existence of statistically significant difference between values with *p* < 0.05; NS, non-significant. BMI, body mass index; PASI, psoriasis area and severity index; TGs, triglycerides; HDL, high-density lipoproteins; LDL, low-density lipoproteins; CRP, C-reactive protein; ALT, alanine transaminase; AST, asparagine transaminase; PSQI, Pittsburgh Sleep Quality Index; RLS, restless legs syndrome. Data are shown as Mean ± SD for normal distributed values or median (full range) for skewed distribution values.

**Table 4:** Comparison of baseline data, laboratory parameters, and values assessing the quality of sleep between topical and systemic treatment subgroups.

Parameter	Topical Treatment *n* = 25	Systemic Treatment *n* = 35
BMI	25.27 (17.01–42.1)	28.81 (17.56–41.21) NS
PASI Before	*12.14 ± 6.41*	*15.63 ± 7.2* NS
ALT	19.5 (7–92)	16.5 (6–98) NS
AST	24.5 (10–41)	22 (10–100) NS
Cholesterol	163.7 ± 40.51	152.7 ± 39.21 NS
HDL	46.75 ± 17.58	42.13 ± 11.36 NS
LDL	43 (24–88)	39 (25–197) NS
TG	**116.5 ± 46.72**	145.4 ± 69.2 *
Uric acid	5.3 (3.2–11.17)	6.2 (3.3–8.4) NS
Glucose	90.73 ± 19.96	94.35 ± 33.2 NS
CRP	*3.31 (1.03–46.2)*	*4.44 (0.66–91)* NS
PSQI	**7 (1–17)**	9 (3–18) **
Subjective sleep assessment	**3.615 ± 1.17**	2.971 ± 1.36 *
STOP BANG	1.654 ± 0.85	1.85 ± 0.78 NS
RLS diagnosis	8/17	15/16 NS
RLS Scoring	**16.88 ± 6.71**	21.87 ± 6.03 *

*/**—means the existence of statistically significant difference between values treatment with *p* <0.05; < 0.01, respectively. *Curved font* means the existence of trend. NS, non-significant. BMI, body mass index; PASI, psoriasis area and severity index; TGs, triglycerides; HDL, high-density lipoproteins; LDL, low-density lipoproteins; CRP, C-reactive protein; ALT, alanine transaminase; AST, asparagine transaminase; PSQI, Pittsburgh Sleep Quality Index; RLS, restless legs syndrome. Data are shown as Mean ± SD for normal distributed values or median (full range) for skewed distribution values.

**Table 5:** Variables independently associated with the RLS diagnosis in psoriatic patients.

Parameters	|t|	*p* Value	*p* Value Summary
Total cholesterol	**2.899**	**0.0338**	*
Glucose	**3.227**	**0.0233**	*
CRP	**3.481**	**0.0176**	*
PSQI	**2.666**	**0.0446**	*

*—means the existence of statistically significant difference between values treatment with *p* < 0.05; CRP, C-reactive protein; PSQI, Pittsburgh Sleep Quality Index; RLS, restless legs syndrome. Variables included (AST, ALT, HDL, LDL, TG, Total cholesterol, glucose, uric acid, CRP, PSQI, DLQI, StopBang Score, RLS).

## Data Availability

Data is available at Corresponding Author upon reasonable request.
